# Retraction Note: RNA m^6^A methylation regulates the epithelial mesenchymal transition of cancer cells and translation of Snail

**DOI:** 10.1038/s41467-023-43307-x

**Published:** 2023-11-16

**Authors:** Xinyao Lin, Guoshi Chai, Yingmin Wu, Jiexin Li, Feng Chen, Jianzhao Liu, Guanzheng Luo, Jordi Tauler, Jun Du, Shuibin Lin, Chuan He, Hongsheng Wang

**Affiliations:** 1 Guangdong Key Laboratory of Chiral Molecule and Drug Discovery, School of Pharmaceutical Sciences, Sun Yat-sen University, Guangzhou, Guangdong 510006 China; 2 MOE Key Laboratory of Gene Function and Regulation, School of Life Sciences, Sun Yat-sen University, Guangzhou, Guangdong 510275 China; 3 Department of Chemistry, Department of Biochemistry and Molecular Biology, and Institute for Biophysical Dynamics, The University of Chicago, 929 East 57th Street, Chicago, IL 60637 USA; 4 MOE Key Laboratory of Macromolecular Synthesis and Functionalization, Department of Polymer Science and Engineering, Zhejiang University, Hangzhou, Zhejiang 310027 China; 5 Department of Rehabilitation Medicine, Center for Translational Medicine, The First Affiliated Hospital, Sun Yat-sen University, Guangzhou, China

Retraction to: *Nature Communications* 10.1038/s41467-019-09865-9, published online 06 May 2019

The authors have retracted this article due to image errors across the figures, and some of the data lacking biological repeats, which calls into question the reliability of the data and the conclusions. In particular, there are errors in the immunohistochemistry images in Figure 6A, invasion assay images in Figure 1C, western blots (Figure 5D and Supplementary Figure 2F) and would healing assay images (Supplementary Figure 1B). The authors would like to apologize for any inconvenience caused to the community.

Xinyao Lin, Guoshi Chai, Yingming Wu, Jiexin Li, Feng Chen, Jianzhao Liu, Guanzheng Luo, Jordi Tauler, Shuibin Lin, Chuan He and Hongsheng Wang agree to this retraction. Jun Du has not responded to any correspondence from the editor or publisher about this retraction.

